# Micromanagement in clinical supervision: a scoping review

**DOI:** 10.1186/s12909-023-04543-3

**Published:** 2023-08-09

**Authors:** Jihyun Lee, Solmoe Ahn, Marcus A. Henning, J. M. Monica van de Ridder, Vijay Rajput

**Affiliations:** 1 Department of Dental Education & Dental Research Institute, School of Dentistry, Seoul National University, 101 Daehak-ro, Jongno-gu, Seoul, 03080 South Korea; 2 Dental Research Institute, School of Dentistry, Seoul National University, Seoul, South Korea; 3 Centre for Medical and Health Sciences Education, Faculty of Medical and Health Sciences, The University of Auckland, Auckland, New Zealand; 4 College of Human Medicine, Michigan State University, Michigan, USA; 5 Department of Medical Education, Dr. Kiran C. Patel College of Allopathic Medicine, Nova Southeastern University, Florida, USA

**Keywords:** Micromanagement, Clinical supervision, Health professions education (HPE)

## Abstract

Micromanagement in clinical supervision in health professions education generally refers to supervision characterized by unproductive excessive control and attention to detail. It can affect autonomy, competence, well-being of learners, teamwork, and ultimately patient care. Despite its potential negative impact on learners and patients, no comprehensive review of this phenomenon has been conducted. This scoping review aims to explore the breadth of extant literature concerning micromanagement in clinical supervision in health professions education and map the body of research on the topic. We followed the Preferred Reporting Items for Systematic Reviews and Meta-Analysis﻿: Extension for Scoping Review (PRISMA-ScR). We searched eight databases, and the final review and analysis comprised 12 articles that examined micromanagement in clinical supervision across health professions education. Micromanagement was conceptualized as ineffective supervisory practices such as undue scrutiny, excessive control, domination, and ineffectual leadership. Conversely, alternatives to micromanagement included entrusting or granting autonomy, coaching for independent practice, and providing effective supervision and leadership. Overall, micromanagement was attributed to individual behavioral and personality factors, such as distrust, perfectionism, self-conviction, and low self-esteem. The consequences of micromanagement included inadequacies in professional development and well-being of trainees and patient care, and organizational dysfunction. Suggested solutions included entrusting or empowering trainees with encouragement and clear communication, open communication efforts by trainees, organizational management for quality supervision, and faculty’s valuing both clinical and educational goals. Current literature on micromanagement—in the context of clinical supervision in health professions education—was found to be sparse, implying a need for more rigorous research and discourse on this understudied area. The findings can be used to recognize, solve, and prevent the prevalent, and often unrecognized, phenomena of micromanagement, which may improve clinical supervision, the professional development of trainees and faculty, organizational management, and ultimately patient care.

**Supplementary Information:**

The online version contains supplementary material available at 10.1186/s12909-023-04543-3.

## Introduction

High-quality clinical supervision is vital to the development of competent medical practitioners, and excellent patient care. Though non-existent or limited supervisory input may affect trainee learning and patient safety [1], the negative impacts of excessive supervision, or micromanagement, may also reduce the benefits of clinical supervision [2, 3]. Most academic healthcare organizations require clinical supervision, reflecting the belief that careful guidance can help trainees develop into independent professional decision makers and competent clinicians. At the same time, there is little empirical validation or theoretical foundation underlying such supervisory practices [4].

A few studies have shed light on some of the components of high-quality clinical supervision in health professions education (HPE). Busari and colleagues [5] reported on trainees’ views of “good” and “poor” supervision. Overall, trainees felt that effective (good) supervisors provided clear explanations of their clinical opinions, gave them autonomy to enhance their experience and competence, and allowed them to engage in self-directed learning. Conversely, less skillful (poor) supervisors showed deficiencies in coaching, including ineffective communication and micromanagement that undermined trainees’ autonomy and compromised their learning and, accordingly, patient safety (e.g., van de Ridder et al. [6]). Interestingly, studies suggest that too much supervision or micromanagement is more common than not enough supervision in medical education practices [1, 5].

Micromanagement can be referred to as an inappropriate method employing excessive clinical supervision. Micromanagement engenders the management of personnel using excessive control or attention to detail. Exerting an excessive level of control denotes that it goes beyond a generally accepted level of input and often culminates in negative consequences. Studies report that micromanagement can have negative influences on medical training and patient care as it creates an unsafe learning environment, harms the learners in their learning, and depletes confidence in future independent clinical practice [2, 3]. In general, micromanagers may appear to be well intended, and in fact, are seldom aware that their behavior has negative effects on a trainee’s motivation, autonomy, competence, well-being, team-work, and patient care. Among the three functions of clinical supervision that Proctor’s model represents — managerial, educational and supportive— [7], the focus of micromanagement is perceived to be excessively monitoring performance, rather than providing education and support. However, recent studies have reported that micromanagement, resulting in improper intensive supervision, did not improve patient safety and outcome [8, 9].

Though all levels of learners (students, interns, residents, and fellows) see micromanagement as problematic [6], the phenomenon has received little attention in HPE. The concept and practice have gone largely unattended, and related issues, such as validly defining micromanagement within clinical care, understanding why it happens and what it brings about, examining consequences, and proposing solutions for the problem, have not been fully explored. Even in the business literature, only a few strategies are proposed for overcoming micromanagement [3, 10–12].

Establishing a theoretical basis for effective clinical supervision in medical settings would go a long way toward preventing the micromanagement of trainees. This scoping review aims to explore the breadth of the available micromanagement literature with reference to clinical supervision across HPE. This work aims to contribute to refining practices related to educating independent competent physicians and enhancing quality patient care.

## Method

We conducted this study using the scoping review method to provide an overview of research available on micromanagement in clinical supervision. We followed the Preferred Reporting Items for Systematic Reviews and Meta-Analysis: Extension﻿ for Scoping Review (PRISMA-ScR) [13]. We chose to conduct a scoping review rather than a systematic review because of the differences in their goals and methods. While a systematic review focuses clearly defines research questions by synthesizing evidences from best available empirical studies, a scoping review broadly addresses defined research questions by exploring breadth instead of depth of the available studies and identifying gaps within the research topic [14]. Thus, a scoping review was more appropriate for our purposes since it would provide a map or a snapshot of the body of research on micromanagement in HPE. Further, our preliminary exploration showed that terms, concepts, and research designs used in relevant articles were diverse and inconsistent, making it difficult to aggregate or weigh evidence. Thus, we saw this scoping review as potentially informing future systematic reviews by providing an overview of the scope of current research and descriptive summaries, and identifying research gaps.

### Search strategy

With the help of two qualified librarians, we searched eight databases judged to be the most relevant to our topic (Web of Science, Scopus, ScienceDirect, Pubmed, PsycINFO, Embase, CINAHL and ﻿ERIC). In addition, we conducted a hand search at Google Scholar in order to cover the breadth of the healthcare professions education literature. We performed all database searches on February 22, 2021 using search terms in the title, abstract, or keyword of articles according to the PCC (Population, Concept, Context) framework [13]: (a) health professions (Population); (b) micromanagement (Concept) and (c) education (Context). Full search algorithms for each database can be found in Additional file 1. Because no date range was set, all related published literature was included in the search.

### Selection of sources of evidence

All authors independently assessed the title and abstract screenings and reviewed the full-texts of all papers against the eligibility criteria. Discrepancies were resolved through consensus-driven meetings focused on determining the suitability of the articles for final review. The inclusion and exclusion criteria are presented in Table 1.

**Table 1:** Inclusion and exclusion criteria

Criterion	Inclusion	Exclusion
Time period	Not limited to any publication date	
Language	English	Not English
Type of article	Peer reviewed journal publication	Non-peer-reviewed articles
Type of study	All types (e.g., original research, commentary, letter to editor, perspective)	
Accessibility	Full-text available or accessible through library loan	Full-text not accessible
Discipline	Health professions education (HPE)	Other disciplines such as biology, policy, law, business, game, or engineering
Study focus	Micromanagement as clinical supervision across HPE	Micromanagement is mentioned but not the main focus of the paper
Context	All contexts of HPE if there were clinical supervision.	Not related to HPE; micromanagement in the context of patient care or health care organization management

### Data extraction and synthesis

We extracted two types of data: study features and findings. We extracted the study features of independent articles by year of publication, country where the study was conducted, health professions discipline (medical, dental, or nursing), journal/section, workplace relationships, research method, and sample size.

We extracted the findings of the articles following a four-step procedure [15]. First, we produced short summaries of each study. Second, we identified key items in the summaries, and developed a standard category template consisting of conceptualization, potential counter-balancing concepts, reasons, consequences, and possible solutions for micromanagement based on our scoping review aims. Third, we analyzed and sorted the items in each category of our template, resulting in groups of main and sub-themes. For consequences and solutions, we adopted the theoretical framework for clinical supervision developed by Rothwell et al. [16]: professional development, organizational development, and patient services. Finally, we collated the charted information, and synthesized the template into a graphical chart in order to unearth the true characteristics of all reviewed articles. We jointly and iteratively refined the interim and final outputs of the data extraction until we arrived at consensus.

## Results

**Fig. 1:**
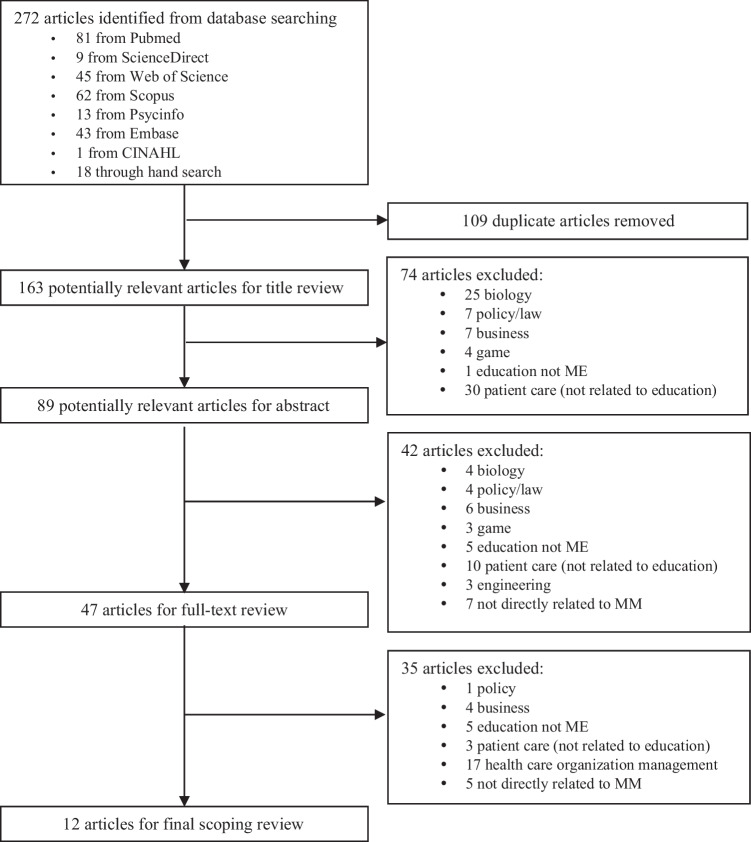
PRISMA flow diagram for a scoping review of micromanagement in clinical supervision

We identified a total of 272 articles from the 8 academic databases and an additional hand search, and then eliminated 109 duplicate articles. We screened 163 potentially relevant articles by title review, resulting in 74 articles being eliminated based on their titles, with 89 potentially relevant articles remaining for abstract review. After we reviewed these 89 abstracts thoroughly, we excluded 42 articles. We retrieved the remaining 47 articles for full-text review, after which we excluded 35 more with consensus. Details of the exclusion process along with reasons for exclusion are presented in Fig. 1.

### Features of reviewed articles

The final analysis consisted of 12 articles with seven distinguishing features: (1) publication year, (2) country of the first author’s institution, (3) health profession discipline (4) journal name and section, (5) workplace relationship, (6) research method, and (7) sample size, as shown in Table 2.

**Table 2:** Features of reviewed studies

Study/ Publication Year	Country of First Author’s Affiliation	Discipline	Journal Name	Section	Workplace Relationship	Research Method	Sample Size
Campbell, 2010 [17]	USA	Nursing	Nursing Management	Leadership Q&A	Manager Nurse-Nurse	-	-
Carbo and Huang, 2019 [18]	USA	Medicine	The Clinical Teacher	Clinical Teacher’s Toolbox	Faculty-Resident	-	-
Cleary et al., 2015 [2]	Australia	Nursing	Issues in Mental Health Nursing	Original Research	Manager Nurse-Nurse	Commentary based on Narrative Review	-
Crockett et al., 2019 [19]	USA	Medicine	BMC Medical Education	Original Research	Faculty-Resident	Qualitative (Focus Group Interview)	59 resident physicians
Emberton, 2020 [20]	USA	Medicine	The Permanente Journal	Commentary	Doctor-Medical team	-	-
Farnan et al., 2009 [21]	USA	Medicine	The Association of Professors of Medicine (APM)	APM Perspectives	Attending Physician-Physician in training	Qualitative (individual interview) & Quantitative (survey)	90 (46 trainees/44 attending physicians)
Kerfoot, 1998 [22]	USA	Nursing	Nursing Economics	On Leadership	Manager Nurse-Nurse Doctor-Nurse	-	-
Levin, 2016 [23]	USA	Dentistry	The Journal of the American Dental Association	Q & A	Dentist-Dental team	-	-
Ranji, 2020 [24]	USA	Medicine	The Journal of the American Medical Association, JAMA	Opinion	Faculty-Resident	-	-
Reynolds, 2012 [25]	USA	Dentistry	Journal of Michigan Dental Association	Vignette	Dentist-Dental team	-	-
Santen et al., 2019 [26]	USA	Medicine	Western Journal of Emergency Medicine	Original Research	Faculty-Resident	Qualitative (Focus Group Interview)	4 (2 faculty and 2 residents)
Van de Ridder et al., 2020 [6]	USA	Medicine	Journal of Graduate Medical Education	To the Editor	Faculty-Trainees (on every level)	-	-

Publication year: Although we did not limit the publication year in the search stage, all the final articles were published between 2009 and 2020, except for one article published in 1998. This result shows the degree to which academic interest in the area has recently emerged. Country of the first author’s institution: For the vast majority of articles (*n* = 11, 91.7%), the educational institution of the first author was located in the United States [6, 17–26], with one in Australia [2]. Health profession discipline: Over half of the articles (58.3%, *n* = 7) concerned medicine [6, 18–21, 24, 26], 25% (*n* = 3) were related to nursing [2, 17, 22], and 16.7% (*n* = 2) referred to dentistry [23, 25]. Journal: The 12 publications were published in 12 different journals, 4 (33.3%) were published in HPE-related journals [6, 18, 19, 21], 3 (25%) were published in medical or dental association journals [23–25], and 4 articles were published in non-HPE journals. Research method/Journal section/Sample size: Four of the 12 publications (33.3%) were original research [2, 19, 21, 26], which consists of two qualitative methods, one mixed method; and one narrative literature review. Two articles conducted focus group interviews [19, 26], one with 59 physicians [19], and one with two faculty members and two residents [26]. Another study used both individual interviews and surveys with 46 trainees and 44 attending physicians [21], while another incorporated a narrative review of 26 papers on micromanagement among mental health nurses [2]. Other articles were anecdotal commentaries with varied section titles [6, 17, 18, 20, 22–25]: Commentary (20), Opinion [24], To the Editor [6], On leadership [22], Clinical teacher’s toolbox [18], and Leadership Q & A (Questions and Answers) [17]. Workplace relationship: The majority (58.3%, *n* = 7) of the key workplace relationships were medical faculty and trainee/residents [6, 18–21, 24, 26]. Other relationships were between manager nurse and trainee nurse 25% (*n* = 3) [2, 17, 22]; and dentist and trainee dental team 16.7% (*n* = 2) [23, 25].

### Synthesis of findings of reviewed articles

We identified five categories from the articles we reviewed: (1) conceptualization of micromanagement, (2) counter-balancing concepts of micromanagement, (3) reasons/influencing factors, (4) consequences, and (5) possible solutions. We sorted each consequence and solution in terms of professional development, organizational development, and patient services, according to the framework for clinical supervision developed by Rothwell and colleagues [16]. Table 3 delineates five categories, main themes and their sub-themes, and provides representative phrases and their sources. Figure 2 illustrates the interconnections between main themes.

**﻿Fig. 2:**
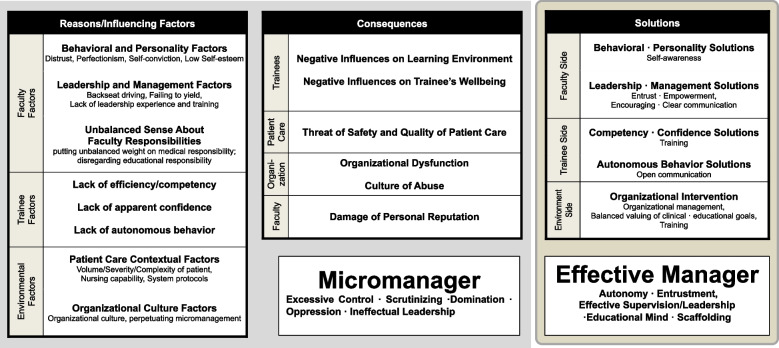
Summary diagrams on reasons/affecting factors, consequences, and possible solutions of micromanagement

**Table 3:** Summary of reviewed studies

Topics	
Concepts	**Excessive Control** [2, 6, 18, 22, 26] - exercising tight/excessive control over trainees - sense of need to control everything **Scrutinizing** [2, 6, 18, 22, 23] - unnecessary attention to every last detail - closely monitoring the minutiae of work practices **Domination/Oppression** [18, 20–23] - full domination; being autocratic; forcing conformism - cannot work through others; not allowing trainees to make autonomous decisions **Ineffectual Leadership** [20] - being autocratic; emphasizing conformity rather than organizational learning
Potential Counter-balancing Concepts	**Autonomy/Entrustment** [2, 6, 18–22, 24, 26] - granting trainee’s an appropriate level of autonomy; - being aware of what micromanagement brings - entrusting trainees to care for patients autonomously; Entrustable professional activities - promoting trainee engagement/trainee’s sense of responsibility for patients **Effective Supervision** [18, 19, 24] - appropriate/consistent/effective supervision **Effective Leadership/Leading** [2, 19, 20, 22] - having a team of specialists work; working through others **Educational Mind/Scaffolding** [6, 18–20, 24, 26] - promoting trainee’s independent practice and organizational learning; - developing learners’ progress towards the ultimate goal of independent practice; - trainees to actively hone their own skills - individual coaching; mentoring; scaffolding
Reasons/ Affecting Factors	**FACULTY FACTORS** BEHAVIORAL AND PERSONALITY FACTORS **Distrust** [2, 6, 17, 18, 23, 26] - trouble trusting others; extreme irritation when trainees make even the smallest of decisions without first consulting them; believing that only they can do the job correctly; ownership of patients; lower threshold to intervene with trainees - personal insecurities **Perfectionism** [2, 6, 22, 23, 26] - need to be perfect in the eyes of others; pressures to meet key performance indicators; obsessive high-achiever personality - fear of failure; avoidance of errors; risk aversion; nervousness about either overall practice level or trainee’s performance; **Self-conviction** [17, 22, 26] - being more professionally confident; judging themselves superior - arrogance and grandiosity **Low Self-esteem** [22, 26] - strive to overachieve to demonstrate their worth - self-doubt; lack of confidence with their own skills LEADERSHIP AND MANAGEMENT FACTORS **Backseat Driving** [19] - not leaving work area, imposing personal management style **Failing to Yield** [19] - predetermining course of action; changing plans without alerting trainees **Lack of Leadership Experience and Training** [2, 17, 22, 23] - recently moved into the ranks of leadership from a prior non-supervisory position; inexperience - no leadership training UNBALANCED SENSE ABOUT FACULTY RESPONSIBILITIES **Unbalanced Commitment to Patient Care and Clinical Education** [26] - putting undue weight on clinical care and responsibility - disregarding educational responsibility
	**TRAINEE FACTORS** **Lack of Efficiency/Competency** [26] - the year (level) of training; clinical experiences - trainee’s performance in terms of effectiveness and efficiency **Lack of Apparent Confidence** [26] - lack of authenticity in self-confidence; preconceived view of the trainee **Lack of Autonomous Behavior** [26] - lack of self-determination and autonomous behavior
	**ENVIRONMENTAL FACTORS** PATIENT CARE CONTEXTUAL FACTORS **Volume/Severity/Complexity of Patient Care** [21, 25, 26] - patient volume (how busy was department); - the acuity/severity of the patient; - high complexity/uncertainty of problem or task; - socio-medical issues of patient/family **Nursing Capability** [26] - number, skills or experience of the nursing staffs **System Protocols** [25, 26] - some case requiring faculty presence or higher precision ORGANIZATIONAL CULTURE FACTORS **Organizational Culture Perpetuating Micromanagement** [2, 24, 26] - culture of high performance; measuring quality metrics; - culture of close supervision; - tight regulations of duty hours
Consequences	***Professional Development Perspective﻿*** **CONSEQUENCES FOR TRAINEE** **Negative Influences on Learning Environment** [6, 21, 24] - loss of educational development and self-confidence; preventing trainees from fully developing their own clinical skills; restricting trainee autonomy and competence; - loss of enthusiasm and creativity; generating a sense of trainee’s apathy **Negative Influences on Trainee’s Wellbeing** [6, 21, 24] - trainee fatigue/burnout; - trainees’ increased resentment and cynicism; - threatening trainees’ psychological, emotional and cognitive safety; - poor health outcomes of trainees
	**CONSEQUENCES FOR FACULTY** **Damage to Personal Reputation of supervisor** [2]
	***Patient Service Perspective*** **CONSEQUENCES FOR PATIENT CARE** **Threat of Safety and Quality of Patient Care** [22, 23] - threatening safe patient care; - ineffective patient care; - undermining practice capacity to serve patients
	***Organizational Development Perspective*** **CONSEQUENCES FOR ORGANIZATION** **Organizational Dysfunction** [6, 22, 23, 26] - high staff turnover; decreased job satisfaction; - absenteeism; being laissez faire; - stifling team-members’ enthusiasm and creativity; - preventing team members from contributing to discussions, making initiatives, and being engaged - debilitating team- **Culture of Abuse** [6, 22, 23] - demoralizing team - harming relationship among trainees; bickering among each other - lack of unity within teams; lack of goodwill
Solutions	***Professional Development Perspective*** **FACULTU SIDE** SOLUTIONS FOR FACULTY BEHAVIORAL AND PERSONALITY FACTORS **Self-awareness** [2, 22, 23, 25] - recognizing the tendency of micromanagement and admitting that it is natural; assessing faculty their own ability to work through others effectively; studying the triggers for micromanagement; delineating between support and micromanaging and identifying when to rectify incongruence; planning for gradual improvement Solutions for Faculty Leadership and management factors **Entrust/Empowerment** [19, 21–23, 25] - delegating everything possible; challenging trainees to think and act independently; promoting their decision making; encouraging their patient ownership; - ‘roadside assistance’; back-stage approach to clinical oversight; serving as safety net; - spreading the work load; sharing reward; enjoying success together; learning the power of a team; - development of trust (truthfulness and benevolence) **Encouraging and Clear Communication** [2, 19] - promoting constructive communication styles, e.g., praising abilities; clear communication regarding roles and responsibility **Training** [21, 24] - faculty development or enhancing effective supervisory strategies in clinical care
	***Professional Development Perspective*** **TRAINEE SIDE** SOLUTIONS FOR TRAINEE COMPETENCY AND CONFIDENCE FACTORS **Training** [21] - competency-based education; trainees’ milestone in professional development - trainee education on the importance of seeking supervision in clinical care and recognizing the liability inherent in the clinical decision-making process SOLUTIONS FOR TRAINEE AUTONOMY FACTORS **Open Communication** [2, 17, 20] - open the conversation by focusing trainee’s optimal contribution; asking for feedback and areas for improvement to identify supervisor’s concern; gently describing the impact of micromanagement; team members being open in their support of team goals and priorities
	***Organizational Development Perspective*** **ENVIRONMENT SIDE** ORGANIZATIONAL INTERVENTION **Organizational Management** [2, 19, 24] - reducing work load pressure on ‘attendings’; - being sensitive to team dynamics and hierarchy; - providing support systems and ongoing assistance for supervisors and trainees; - redefining and evaluation of quality supervision **Balanced Valuing of Clinical and Educational Goals** [19] - modernizing the organizations to achieve two equally important goals of improving the quality of care and enhancing residents’ education **Training** [6, 18] - organizational training on mentoring, coaching, autonomy and trust building

#### Conceptualization of micromanagement

The articles contained ﻿concepts associated with micromanagement in clinical supervision. The most common were excessive control (feeling the need to exert control tightly over trainees) [2, 6, 18, 22, 26] and scrutinizing (paying attention to and monitoring every last detail) [2, 6, 18, 22–24], followed by domination or oppression (being autocratic, forcing conformism, and not allowing trainees to make autonomous decisions) [18, 20–23]. In one article, micromanagement was conceptualized as ineffectual leadership (trainees learn from the established hierarchy, not from a “teacher”) [20]. Other articles discussed potential counter-balancing concepts﻿ of micromanagement; the most common being autonomy or entrustment (entrusting trainees to care for patients with appropriate level of supervision and autonomy) [2, 6, 18–22, 24, 26], followed by educational mindset (mentoring, coaching, or scaffolding so that trainees progress toward independent practice) [6, 18–20, 24, 26]. Contrary to ineffectual leadership, effective supervision [18, 19, 24] and leadership [2, 19, 20, 22] were presented as concepts opposite from micromanagement.

#### Reasons/influencing factors in micromanagement

The articles we reviewed proposed a variety of reasons or factors influencing micromanagement in clinical supervision. We classified these as: faculty, trainee, or environmental factors.

Within the category of faculty factors we identified three sub-factors: (1) behavioral and personality factors [distrust [2, 6, 17, 18, 23, 26], perfectionism [2, 6, 22, 23, 26], self-conviction [17, 22, 26] and low self-esteem [22, 26]; (2) leadership and management factors [“backseat driving” [19], failing to yield [19], and lack of leadership experience and training [2, 17, 22, 23], and (3) unbalanced sense of responsibility (putting more weight on the faculty’s role in patient care while relatively disregarding the role of clinical education) [26]. Trainee factors included: (1) lack of competency or efficiency [26]; (2) apparent lack of confidence [26]; and (3) lack of autonomous behavior [26]. There were two environmental sub-factors: (1) patient care contextual factors and (2) organizational culture factors. Patient care contextual factors concerned volume/severity/complexity of patient care [21, 25, 26], nursing capability [26], and system protocols that require faculty presence or higher precision [25, 26]. Organizational culture factors that perpetuate micromanagement (high performance culture, close supervision, and tight regulations) [2, 24, 26].

#### Consequences of micromanagement

We delineated and classified the variety of consequences of micromanagement into four groups, including the consequences for: (1) professional development of trainee; (2) patient service; (3) organizational development; and (4) faculty (supervisor).

The consequences for trainees’ professional development were: (1) negative influence on learning environment (trainee loss of educational development and self-confidence, loss of enthusiasm and creativity) [6, 21, 24] and (2) a negative influence on trainee wellbeing (trainee fatigue or burnout and increased resentment, threats to psychological and physical health) [6, 21, 24].

Consequences for patient service referred to threats to the safety and quality of patient care [22, 23]. Micromanagement may result in less effective training for learners, thus influencing the effectiveness of patient care and undermining practice capacity. From the organizational development perspective, consequences for organizations were (1) organizational dysfunction [6, 22, 23, 26] due to high staff turnover, decreased job satisfaction, absenteeism or stifled enthusiasm, and (2) a culture of abuse [6, 22, 23] that demoralizes trainees, harms relationships within a team, and debilitates team unity. Finally, as a consequence for faculty, damaged personal reputation was highlighted [2].

#### Suggested solutions

Overall, the studies reported solutions for micromanagement in terms of faculty perspective, trainee perspective, and organizational development perspective. The solutions tended to correspond to the reasons/influencing factors for micromanagement. From a professional development perspective of supervisors, self-awareness [2, 22, 23, 25] of tendencies toward and triggers of micromanagement were solutions suggested in multiple articles. Other steps similarly relied on introspection and self-assessment, such as of the ability to work through others effectively and their own triggers for micromanagement. Other recommendations involved supervisors understanding the differences between support and micromanagement and knowing when to rectify incongruence, and planning for gradual improvement. Entrustment and empowerment [19, 21–23, 25] referred to actions that would promote trainees’ independent thinking and acting, perhaps by trying to take a back-stage approach to clinical oversight. Clear communication about trainees’ roles and responsibility, and faculty’s expectations [2, 19], were also perceived as essential, as well as training in leadership and supervisory strategies Training [21, 24]. From the perspective of trainees’ professional development, both clinical training to improve competence and efficiency, and trainee training to seek effective supervision together with a recognition of their liabilities as clinical caregivers were recommended [21], along with open communication with faculty [2, 17, 20] in order to identify concerns and meet their own expectations of and that of their supervisor.

Suggestions linked to changes in the environment focused on enhancing organizational management through such actions as reducing the performance pressure or the provision of support systems that would enable quality supervision [2, 19, 24]. Balancing clinical and educational goals also was mentioned [19], as well as organizational training, mentoring, coaching, and autonomy building [6, 18].

In short, the most frequently addressed concept in relevant studies of micromanagement was scrutinizing (*n* = 6, 50.0%) [2, 6, 18, 22–24]. In contrast, autonomy or entrustment (*n* = 9, 75.0%) [2, 6, 18–22, 24, 26] were the most frequently mentioned contrasting concepts. The most mentioned reasons for the perpetuation of micromanagement were faculty’s behavioral and personality dimensions (*n* = 7, 58.3%), among which distrust was the number one reason (*n* = 6, 50%) [2, 6, 17, 18, 23, 26]. In terms of consequences, the most common concern was organizational dysfunction (*n* = 4, 33.3%), with the foremost solution focusing on changes in supervisory leadership and management strategies geared toward entrusting and empowering trainees (n = 5, 41.7%).

## Discussion

This scoping review explored the literature pertaining to micromanagement in clinical supervision in ﻿health professions education. The key messages are: (1) Micromanagement in clinical supervision was conceptualized as scrutinizing, excessive control, domination and ineffectual leadership; (2) it is attributed to faculty members’ behavioral and personality factors foremost; (3) the consequence of such micromanagement likely impacts trainees’ professional development and well-being, patient care, and organizational dysfunction; (4) micromanagement can be mitigated by solutions such as faculty’s entrusting or empowering trainees with clear encouraging communication, open communication efforts from trainees, organization management for quality supervision, and valuing both clinical and educational goals; and (5) more research, based on a higher quality of evidence, is needed to understand and discuss micromanagement in clinical supervision. These five key messages will be discussed in turn.

Our scoping review suggests that in the field of HPE, micromanagement in clinical supervision has negative connotations, as evidenced by associated features like scrutinizing, excessive control, domination, and ineffectual leadership. Conversely, alternatives to micromanagement were essentially positive, including entrustment or granting autonomy, coaching for trainees’ independent practice, and effective supervision and leadership. Nonetheless, supervisory practices associated with micromanagement mostly engender negative perceptions and, as such, the field of medicine seems more tolerant of this approach to clinical training than fields outside medicine, such as organizational management, public administration, and political science [27]. In the field of organizational management, Peter Drucker’s 1946 work on democracy in management (decentralizing and delegating more authority to employees) and Douglas McGregor’s 1960 Theory X manager (a manager who is poor at proper delegating), criticize micromanagement as a strong disrupter of organizational life and an organizational pathology [28]. However, in the health professions, the perception of micromanagement is still controversial due to the criticality of patient safety although it was reported that micromanagement does not improve patient safety and outcomes [8, 9].

Given the greater tolerance for supervisory micromanagement in medical fields [27], it is reasonable to ask, what exactly is the problem with scrutinizing, i.e., monitoring every last detail or a detail-oriented faculty? Some aspects of performance by health professionals are crucially important— such as those linked to patient safety, performance and professional expertise—and may be seen as justifying ‘over-management’. In fact, among physicians there has been an implicit understanding that detail-oriented, enhanced supervision is both good and necessary [29, 30]. Practices that promote trainees’ autonomy and empowerment stand in contrast to these perceptions, consistent with our finding that the solutions to micromanagement most commonly mentioned in the reviewed articles were supervision entrustment and trainee empowerment. For clinical supervisors, it is sometimes difficult to know when a trainee is ready for unsupervised independent patient care, especially when the evidence is inconclusive, and the opinions of the supervisor and trainee differ [31]. In such cases, the Entrustable Professional Activity (EPA) can help in making such decisions [32]. EPAs are units of practice that medical trainees have to master and that supervisors must trust them to perform adequately before they complete their training. The EPA lists professional tasks and proficiencies at five levels: having limited knowledge, acting under close supervision, acting under supervision on call, acting independently, and supervising others [32]. In terms of when clinical supervisors who micromanage will feel comfortable granting autonomy to a trainee, the use of the EPA-based assessment may be a reasonable solution.

Our review revealed that in the vast majority of studies, micromanagement was associated with individual supervisor factors, particularly behavioral and personality factors, when compared with trainee and environmental factors. Factors such as acute clinical context or lack of trainee clinical competency also were seen as influencing micromanagement behavior, but to a much lesser degree. This suggests that supervisor perceptions of trainee ineffectiveness should not justify micromanaging trainees, but rather highlight the need for scaffolding that equips the trainee to reach a prescribed level of competence. This finding is inconsistent, however, with that of Sterkenburg et al. [31], who investigated factors affecting supervisor entrustment of trainees. They found that entrustment was most influenced by trainee factors, followed by faculty and contextual factors. This perceptual and hierarchical discrepancy to micromanagement may be due to different interpretations of clinical supervision. Some supervisors believe the purpose of clinical supervision is to facilitate the delivery of services to patients and monitor trainee performance, which is termed as managerial supervision [33]. Others see supervision as a vehicle for supporting the professional development of trainees [16]. Having a mutually agreed purpose of clinical supervision may be one of the keys for reducing ambiguity.

The reviewed literature revealed a number of adverse consequences of micromanagement in clinical supervision, including trainees’ educational loss and threats to their psychological and physical health, threats to the safety, efficiency and capacity for patient care, and organizational dysfunction. Importantly, in articles where trainees were quoted, they stressed a sense of helplessness [26]. One of the most frequently mentioned consequences of micromanagement was organizational dysfunction, including high staff turnover, decreased job satisfaction, absenteeism, and the diminished enthusiasm of team members—all bringing about threats to the safety, efficiency, and capacity of patient care. Some articles suggested that micromanagement can temporarily increase productivity [34]. This connection between micromanagement and organizational dysfunction is important, since in most cases organizational dysfunction is linked to a long-term downturn in productivity.

Overall, solutions to micromanagement in clinical supervision centered on the capacity of supervisors to entrust or empower trainees through encouragement and clear communication. More specifically, such solutions involved concrete efforts by supervisors to facilitate open communication with trainees, and organizational management that aims to both support quality supervision and balance clinical and educational goals. Other recommendations included leadership training for supervisors and measures that ensured supervisors promote trainees’ clinical and communication competencies. Among these multiple solutions, supervisor entrustment and empowerment of trainees were most commonly mentioned (41.7%, *n* = 5).

Given our finding that in most studies, micromanagement was attributed to individual supervisor factors, it is no surprise that an important solution involves training and development initiatives for faculty. Promoting self-awareness is a logical starting point for gradual improvement in entrusting or empowering trainees. In addition, supervisors need to know about the demonstrated positive benefits of good clinical supervision, such as trainees’ reduced stress and anxiety, increased resilience, and job satisfaction. Upskilling and increased quality of care also result from effective supervision, which is best provided in an open, supportive, trusted environment that facilitates discussion and reflection on clinical practice [16]. It is also noteworthy that the organizational role can be facilitative when managing the adverse culture of micromanagement to ensure that patient care and educational goals are equally valued and emphasized. One method can optimize collaboration between an educational supervisor (who concerns educational development) and a clinical supervisor (who concerns clinical practice) to reduce the tension between the two important values [16].

This scoping review has several limitations: the sample of relevant articles we identified from academic databases is fairly small, although we searched eight databases. The sample size suggests that micromanagement in clinical supervision is an emerging research area, and points to the appropriateness of a scoping review as a starting point for more rigorous empirically-based research in coming years, such as a systematic reviews, in-depth qualitative analysis, empirical investigations, and cohort studies. From further empirical research, researchers and practitioners can gain a more precise picture of micromanagement in clinical supervision. Although our analysis was inevitably limited to publications written in English, it was noted that USA cases were overwhelmingly included, which resulted in a lack of an international perspective in our study. Future research efforts could incorporate a more inclusive international viewpoint. Additionally, research on supervision in the field of psychology and mental health (e.g. [35, 36].), a field adjacent to health professions could further enrich our understanding and provide additional valuable insights into the dynamics of micromanagement. In addition, the conceptual features of reviewed articles could be evaluated through future research using the consultation exercise of scoping review methodology in order to identify current issues facing key practicing stakeholders [15]. With the advent of a more enhanced evidence-based foundation, more precise and effective processes for managing and ameliorating the effects of micromanagement could be implemented.

## Conclusion

In current literature on micromanagement in clinical supervision, researchers have conceptualized micromanagement, and discussed its reasons/influencing factors, consequences, and solutions. The ineffective and effective supervisory practices associated with micromanagement that we identified in this study have implications for clinical supervisors in health profession educational settings. Such knowledge, as well as insights about alternative supervisory practices, reasons/influencing factors, consequences, and suggested solutions, can be used to recognize, solve, and prevent the prevalent, and often unrecognized, manifestation of micromanagement. In addition, by including the perspectives from supervisors, trainees, organizations, and patients, the findings can be used by health professions educators to develop various approaches to training, learning, and healthcare that best represent the needs of all stakeholders. We hope this review offers a useful springboard for more targeted empirical work and academic discourse around this topic, which could improve the quality of clinical education and patient care.

### Supplementary Information

**Additional file 1.**

## Data Availability

Related data of this study can be available upon request to the corresponding author.
