# Supplementary material for: Micromanagement in clinical supervision: a scoping review
Source: BMC Med Educ. 2023 Aug 9;23:563. doi: 10.1186/s12909-023-04543-3 (PMC10410949; doi:10.1186/s12909-023-04543-3)
Supplement: Supplementary file 1 — Additional file 1. [file 12909_2023_4543_MOESM1_ESM.docx]

**Supplemental Digital Content**

**Search Queries**

**Web of science (n=45)**

(TS=((micromanagement) AND ((clinical OR medic* OR dent* OR nurs* OR "health sciences") OR (supervis* OR school OR student OR learn* OR teach* OR education)))) AND LANGUAGE:(English)
AND DOCUMENT TYPES:(Article)

**SCOPUS (n=62)**

TITLE-ABS-KEY((micromanagement) AND ((clinical OR medic* OR dent* OR nurs* OR "health sciences") OR (supervis* OR school OR student OR learn* OR teach* OR education))) AND (LIMIT-TO ( DOCTYPE,"ar")) AND (LIMIT-TO (LANGUAGE,"English"))

**Embase (n=43)**

micromanagement AND ('clinical'/exp OR clinical OR medic* OR dent* OR nurs* OR 'health sciences' OR supervis* OR 'school'/exp OR school OR 'student'/exp OR student OR learn* OR teach* OR 'education'/exp OR education) AND [article]/lim AND [english]/lim

**ScienceDirect (n=9)**

Title, abstract, keywords:

1 (((micromanagement) AND (clinical OR medical OR dentistry OR nursing OR "health sciences"))

2 ((micromanagement) AND (supervisor OR school OR student OR learning OR teaching OR education)))

**Pubmed Results (n=81)**

((micromanagement)[Title/Abstract] AND ((clinical[Title/Abstract] OR medic*[Title/Abstract] OR dent*[Title/Abstract] OR nurs*[Title/Abstract] OR "health sciences")[Title/Abstract] OR (supervis*[Title/Abstract] OR school[Title/Abstract] OR student[Title/Abstract] OR learn[Title/Abstract] OR teach[Title/Abstract] OR education)))[Title/Abstract] AND ("journal article"[Publication Type]) AND ("english"[Language])

**PsycINFO (via EBSCO) Results (n=13)**

TI ((micromanagement) AND ((clinical OR medic* OR dent* OR nurs* OR "health sciences") OR (supervis* OR school OR student OR learn* OR teach* OR education))) OR AB ((micromanagement) AND ((clinical OR medic* OR dent* OR nurs* OR "health sciences") OR (supervis* OR school OR student OR learn* OR teach* OR education))) OR KW ((micromanagement) AND ((clinical OR medic* OR dent* OR nurs* OR "health sciences") OR (supervis* OR school OR student OR learn* OR teach* OR education)))

Limiters – Full Text; Peer Reviewed Journal; Document Type: Journal Article Language: English

**CINAHL (via EBSCO) Results: 1**

TI (((micromanagement) AND ((clinical OR medic* OR dent* OR nurs* OR "health sciences") OR (supervis* OR school OR student OR learn* OR teach* OR education)))) OR AB (((micromanagement) AND ((clinical OR medic* OR dent* OR nurs* OR "health sciences") OR (supervis* OR school OR student OR learn* OR teach* OR education))))

Limiters - Full Text; Peer Reviewed Journals; Publication Type: Journal Article; Language: English

**ERIC (via EBSCO) Results: 0**

TI ((micromanagement) AND ((clinical OR medic* OR dent* OR nurs* OR "health sciences") OR (supervis* OR school OR student OR learn* OR teach* OR education))) OR AB ((micromanagement) AND ((clinical OR medic* OR dent* OR nurs* OR "health sciences") OR (supervis* OR school OR student OR learn* OR teach* OR education))) OR KW ((micromanagement) AND ((clinical OR medic* OR dent* OR nurs* OR "health sciences") OR (supervis* OR school OR student OR learn* OR teach* OR education)))

Limiters - Full Text; Peer Reviewed Journals; Publication Type: Journal Article; Language: English
